# Supplementary material for: Content-rich biological network constructed by mining PubMed abstracts
Source: BMC Bioinformatics. 2004 Oct 8;5:147. doi: 10.1186/1471-2105-5-147 (PMC528731; doi:10.1186/1471-2105-5-147)
Supplement: Additional File 2 — The original results of the above study (non-essential files are deleted to keep the file size under the limit set by BMC bioinformatics). [file 1471-2105-5-147-S2.bz2 › chilibotAdditionalFile2/dip20/702ID10809785E18035/html/MALG_MALK.html]

 


 **MALG** and **MALK** 
  
Found 36 abstracts in PubMed, retrieved 20.  
 

 What does Google say? 
 PDF only 
| .edu only 

---

**Interactive relationship** (e.g. stimulation, inhibition, etc)

**Neutral relationship**- **MalK**  bound specifically to MalF and  **MalG**  and reconstituted a functional complex.  Ref: 9791180 Mol Microbiol, 1998
- We focused on interactions between the nucleotide binding protein,  **MalK** , and the transmembrane proteins, MalF and  **MalG** .  Ref: 9791180 Mol Microbiol, 1998
- 1998 Nature London 39 03 707 and are interpreted in favor of a role of the signature sequence in activating the hydrolyzing activity of  **MalK**  upon substrate initiated conformational changes in MalF  **MalG** .  Ref: 10561582 Eur J Biochem, 1999
- We used this approach and limited proteolysis with trypsin to show that binding and hydrolysis of ATP, inducing conformational changes in  **MalK** , modulate its interaction with MalF and  **MalG** .  Ref: 9791180 Mol Microbiol, 1998
- To this end, single cysteine residues were introduced into each subunit at several positions and the respective malF and  **malG**  alleles were individually co expressed with each of the  **malK**  alleles.  Ref: 10809785 J Biol Chem, 2000
- We have used LacK as a tool to identify sites of interaction of  **MalK**  with the membrane integral components MalF and  **MalG** .  Ref: 8951813 Mol Microbiol, 1996
- The maltose transport complex of Escherichia coli, a member of the ATP binding cassette ABC superfamily, is made up of two nucleotide binding subunits,  **MalK**  2, which hydrolyze ATP with positive cooperativity, and two transmembrane subunits, MalF and  **MalG** .  Ref: 12093921 Proc Natl Acad Sci U S A, 2002
- These results indicate that a putative helical domain in  **MalK** , encompassing residues 89 140, is crucial for a functional, high affinity interaction with MalF and  **MalG** .  Ref: 8951813 Mol Microbiol, 1996
- The results suggest that residues Ala 85, Lys 106, Val 114, and Val 117 in the helical segment of  **MalK** , to different extents, participate in constitution of asymmetric interaction sites with the EAA loops of MalF and  **MalG** .  Ref: 10809785 J Biol Chem, 2000

**Non-interactive relationship** (e.g. studied together, co-existance, homology, etc.)

- In our studies of the oligomerization of this complex, we have shown that the ATP binding component,  **MalK** , forms a dimer in the absence of MalF and  **MalG** .  Ref: 10037713 J Biol Chem, 1999
- The MTC is made up of two integral membrane proteins, MalF and  **MalG** , and a peripheral membrane protein,  **MalK** .  Ref: 10037713 J Biol Chem, 1999
- The maltose transporter contains two transmembrane subunits, MalF and  **MalG** , and two copies of a nucleotide hydrolyzing subunit,  **MalK** .  Ref: 11150310 J Biol Chem, 2001
- This uncoupled activity was not due to dissociation of the  **MalK**  ATPase subunit from the integral membrane protein MalF and  **MalG**  subunits.  Ref: 10648525 J Bacteriol, 2000
- First, we optimized the yield in purified complex protein by taking advantage of a newly constructed expression plasmid that carries the  **malK** , malF and  **malG**  genes in tandem orientation.  Ref: 12225853 Biochim Biophys Acta, 2002
- We have investigated the proximity of residues in a conserved sequence EAA loop of MalF and  **MalG**  to residues in a helical segment of the  **MalK**  subunits by means of site directed chemical cross linking.  Ref: 10809785 J Biol Chem, 2000
- Analysis of flanking database sequences from M. tuberculosis and M. leprae revealed the presence of malF,  **malG**  and  **malK**  like genes.  Ref: 10817516 Microbios, 2000
- A putative helical domain in the  **MalK**  subunit of the ATP binding cassette transport system for maltose of Salmonella typhimurium MalFGK2 is crucial for interaction with MalF and  **MalG** .  Ref: 8951813 Mol Microbiol, 1996
- Maltose negative mutants fall into two categories with respect to the cellular localization of the  **MalK**  ATPase in the first,  **MalK**  is membrane bound, as in wild type strains, while in the second, it is cytosolic, as in strains deleted in the malF and  **malG**  genes.  Ref: 9214624 EMBO J, 1997
- The binding protein dependent maltose transport system of enterobacteria, a member of the ATP binding cassette ABC transporter superfamily, is composed of two integral membrane proteins, MalF and  **MalG** , and two copies of an ATPase subunit,  **MalK** , which hydrolyze ATP, thus energizing the translocation process.  Ref: 10561582 Eur J Biochem, 1999
- ATPase activity was not due to dissociation of the  **MalK**  subunit from the integral membrane proteins MalF and  **MalG**  but could not be further stimulated by trehalose maltose binding protein TMBP, be it the native protein as isolated from T. litoralis or the soluble engineered protein.  Ref: 11453995 Eur J Biochem, 2001
- The complex, containing one copy each of the integral membrane proteins  **MalG**  and MalF and two copies of the peripheral cytoplasmic membrane protein  **MalK** , interacts with the periplasmic maltose binding protein to efficiently translocate maltose and maltodextrins across the bacterial cytoplasmic membrane.  Ref: 9573205 J Bacteriol, 1998
- The formation of a  **MalK**  dimer in the absence of MalF and  **MalG**  may represent an initial step in the assembly pathway of the MTC.  Ref: 10037713 J Biol Chem, 1999
- Cyclodextrin transport is mediated by the products of the cymE, cymF, cymG, cymD, and cymA genes, which are functionally homologous to the malE, malF,  **malG** ,  **malK** , and lamB gene products of Escherichia coli.  Ref: 9573146 J Bacteriol, 1998
- We report the characterization of TrmB, a protein of 3 00 apparent molecular weight, that is involved in the maltose specific regulation of a gene cluster in Thermococcus litoralis, malE malF  **malG**  orf trmB  **malK** , encoding a binding protein dependent ABC transporter for trehalose and maltose.  Ref: 12426307 J Biol Chem, 2003
- The proteins of this system are LamB in the outer membrane, maltose binding protein MBP in the periplasm, and the proteins of the inner membrane complex MalFGK2, composed of one MalF, one  **MalG** , and two  **MalK**  subunits.  Ref: 9401026 J Bacteriol, 1997
- We incubated purified  **MalK**  with inverted membrane vesicles containing MalF and  **MalG** .  Ref: 9791180 Mol Microbiol, 1998
- The binding protein dependent maltose transport system of enterobacteria MalFGK 2, a member of the ATP binding cassette ABC transporter superfamily, is composed of two integral membrane proteins, MalF and  **MalG** , and of two copies of an ATPase subunit,  **MalK** , which hydrolyze ATP, thus energizing the translocation process.  Ref: 10809785 J Biol Chem, 2000
